# Suppression of colorectal cancer growth: Interplay between curcumin and metformin through DMT1 downregulation and ROS‐mediated pathways

**DOI:** 10.1002/biof.2137

**Published:** 2024-11-28

**Authors:** Hui‐Yen Chuang, Hui‐Wen Chan, Kuang‐Chung Shih

**Affiliations:** ^1^ Department of Biomedical Imaging and Radiological Sciences National Yang Ming Chiao Tung University Taipei Taiwan; ^2^ Division of Endocrinology and Metabolism, Department of Medicine Cheng‐Hsin General Hospital Taipei Taiwan; ^3^ Division of Endocrinology & Metabolism, Tri‐Service General Hospital National Defense Medical Center Taipei Taiwan; ^4^ School of Medicine National Defense Medical Center Taipei Taiwan

**Keywords:** autophagy, colorectal cancer, curcumin, DMT1, ferroptosis, metformin, ROS

## Abstract

The rising incidence of colorectal cancer (CRC) poses significant healthcare challenges. This study explored the therapeutic potential of combined curcumin (CUR) and metformin (MET) treatment in CRC models. Our findings indicate that the combination treatment (COMB) effectively downregulates the expression of divalent metal transporter‐1 (DMT‐1), leading to a reduction in cell proliferation aligned with suppression of the pAKT/mTOR/Cyclin D1 signaling pathway. The COMB increased reactive oxygen species (ROS) production, triggering activation of the NRF2/KEAP1 pathway. This pathway elicits an antioxidant response to manage oxidative stress in CRC cell lines. Interestingly, the response of NRF2 varied between CT26 and HCT116 cells. Moreover, our study highlights the induction of apoptosis and autophagy, as evidenced by upregulations in Bax/Bcl‐2 ratios and autophagy‐related protein expressions. Notably, the COMB promoted lipid peroxidation and downregulated xCT levels, suggesting the induction of ferroptosis. Ferroptosis has been shown to activate autophagy, which helps eliminate cells potentially damaged by the increased oxidative stress. Furthermore, the COMB effectively diminished the migratory ability of CRC cells. In vivo experiments using CRC‐bearing mouse models, the results confirmed the anti‐tumor efficacy of the COMB, leading to substantial inhibition of tumor growth without inducing general toxicity. In conclusion, our study suggests that combining CUR with MET holds promise as a potential option for CRC treatment, with critical mechanisms likely involving ROS elevation, autophagy, and ferroptosis.

## Graphical abstract

Combination of curcumin with metformin shows great tumor inhibition in CRC‐bearing mice, with critical mechanisms likely involving ROS elevation, autophagy, and ferroptosis.

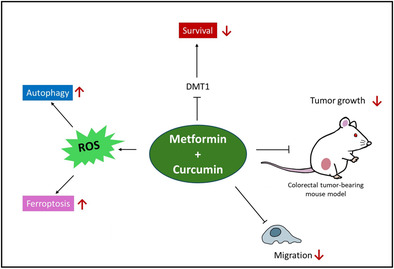

## 1 INTRODUCTION

Colorectal cancer (CRC) ranks as the third most common cancer globally, and both its incidence rate and mortality represent nearly 10% of global cases in 2020.^1^ With its incidence rising and affecting younger populations, its socioeconomic impact deepens.^2^ Epidemiological studies suggest a correlation between systemic iron levels and cancer risk across various types, including CRC.^3^ A recent study reports that the reactive oxygen species (ROS) signaling pathway is associated with cellular iron homeostasis, and its imbalance would intensify ROS production and lipid peroxidation and result in excess cell death.^4^ Upregulation of divalent metal transporter‐1 (DMT1), facilitating intestinal iron absorption, plays a crucial role in colon cancer development.^5^, ^6^ In the mouse model (colon‐selective disruption of DMT1), DMT1 disruption significantly reduces tumor number, burden, and cell proliferation.^6^ As the mental ions typically act as enzyme cofactors, ensuring enzymes function at their maximum catalytic activity; therefore, targeting DMT1 presents a promising anti‐cancer strategy by modulating different biochemical processes.

Metformin (MET), a primary anti‐diabetic medication, reduces glucose production by hampering mitochondrial respiratory chain function, activating AMP‐activated protein kinase (AMPK), and suppressing gluconeogenesis.^7^, ^8^ Beyond its anti‐diabetic properties, there has been significant interest in its anti‐cancer potential, particularly in breast cancer^9^ and CRC.^10^ MET reduces cancer cell viability by downregulating the AMPK/AKT/mTOR pathway,^11^, ^12^ and induces ROS‐mediated DNA damage and cell death.^13^

Curcumin (CUR), a derivative of turmeric, exhibits anti‐cancer properties in CRC, primarily through Nuclear factor kappa‐light‐chain‐enhancer of activated B cells (NF‐κB) inhibition.^14^, ^15^ Additionally, CUR increases ROS production, activating the KEAP1/NRF2/miR‐34a/b/c signaling cascade and reducing cell migration of CRC cells.^16^ Notably, the studies have revealed an enhancing benefit of CUR on treatment of CRC when combined with other therapies, such as radiotherapy,^17^ chemotherapy^18^ or pan‐erb B inhibitor.^19^

Elevated ROS levels can trigger programmed cell death processes such as apoptosis, autophagy, and ferroptosis; accordingly, enhancing ROS production has been proposed as an effective anti‐cancer strategy.^20^ ROS signaling pathway is also associated with cellular iron homeostasis, and its imbalance would intensify ROS production and iron‐mediated lipid peroxidation and result in excess cell death.^4^ While MET and CUR individually exhibit anti‐cancer abilities in CRC, understanding the therapeutic impact of combination with the two agents on CRC progression needs further exploration. Here, we focused on elucidating the anti‐cancer properties of MET in CRC both in cell culture and tumor‐bearing mouse models and exploring how CUR may modulate cellular responses to MET. Our findings suggest that combining CUR and metformin significantly retards tumor progression by downregulating DMT1 expression, promoting ROS‐induced cell death, and suppressing the migration ability of CRC cells.

## 2 MATERIALS AND METHODS

### 2.1 Drug preparation

CUR (Sigma Aldrich, USA) was dissolved in DMSO to prepare a 100 mM stock solution, while MET (Cayman, MI, USA) was dissolved in PBS to yield a 1 M stock solution. Both solutions were stored at −20°C prior to utilization.

### 2.2 Cell culture

Murine and human CRC cell lines, CT26 and HCT116, were used in this study. Both CT26 and HCT116 cells were cultured in RPMI1640 medium supplemented with 10% fetal bovine serum (Corning), 100 IU/mL penicillin, and 0.1 mg/mL streptomycin (Hyclone). All the cells were maintained in a humidified CO_2_ incubator at 37°C.

### 2.3 Cytotoxicity assay

Cytotoxicity of CUR and MET was assessed using the MTT (3‐(4,5‐dimethylthiazol‐2‐yl)‐2,5‐diphenyltetrazolium bromide) assay. Cells were seeded at a density of 1 × 10^4^ cells per well in a 96‐well plate and incubated overnight before being treated with various concentrations of CUR and/or MET for 24 h. Following treatments, the cells were incubated with MTT (0.5 mg/mL)‐containing medium for 2 h, and absorbance at 570 nm was measured to determine cell viability. The IC50 concentrations obtained were used in the following studies to study the potential effects resulting from the combination of CUR and MET.

### 2.4 Cell proliferation assay

Cells were seeded at a density of 1 × 10^5^ cells per well in 12‐well plates and incubated overnight before receiving different treatments, including vehicle control (CTRL), CUR, MET, or their combination at IC50 concentrations. The drug‐containing medium was replaced with a fresh culture medium after a 24‐h incubation. Cell numbers were counted using a hemacytometer at 0‐, 24‐, 48‐, and 72‐h after treatment.

### 2.5 Wound healing

Cell migration was assessed using the ibidi Culture‐Insert 2 well system (ibidi GmbH, Munich, Germany), with 6 × 10^4^ cells seeded into each well. The culture inserts were carefully removed to create a cell‐free gap in the center after cell attachment. Subsequently, cells were then exposed to a drug‐containing medium, and their migration was continuously monitored for 24 h. Images of the gap were captured using a bright‐field microscope (Leica DMIL 090‐135.001, Wetzlar, Germany) equipped with a digital camera at 40× magnification at specified time points as shown in the Figures. Gap width was measured using ImageJ software at each time point to assess cell migration progression by calculating the percentage change in gap width relative to the initial gap width.

### 2.6 ROS assay

ROS generation induced by treatments was detected by the 2′− 7′‐dichlorodihydrofluorescein diacetate (DCFH‐DA) assay. Briefly, cells were collected at 4 and 24 h. after treatments, washed with PBS, and re‐suspended in 400 μL PBS. Then, 1 μL of a 25 mM DCFH‐DA solution was added to the cell suspension, followed by incubation in the dark for 45 minutes at room temperature. As a positive CTRL, H_2_O_2_ (final concentration = 100 μM) was introduced to the untreated cells before DCFH‐DA staining. The stained cells were applied to slides, covered with coverslips, and sealed. The cell images were captured using a fluorescence microscope (BX61, Olympus) equipped with a CCD camera. The fluorescence signal of DCFH‐DA in each group was quantified by ImageJ to assess the changes in ROS levels resulting from different treatments over time.

### 2.7 Lipid peroxidation

Lipid peroxidation at the 4‐ and 24‐h time points post‐treatment was detected by the LiperFluo assay (Dojindo Molecular Technologies, Inc., Rockville, MD, USA). Experiments were performed following the manufacturer's protocols. Optical density was measured using a Multimode microplate reader (TECAN Infinite 200 PRO, Switzerland).

### 2.8 Western blotting

Cells were harvested to examine the changes in protein expression using Western blotting at the 4‐ and 24‐h time points post‐treatment. The collected cells were lysed using RIPA buffer containing PMSF, and the protein concentrations were determined using the Bradford assay. Equal amounts of proteins were mixed with loading dye and denatured at 95°C for 5 min. 30 μg of total cell lysates were loaded in each well and separated by 8% or 12% Sodium dodecyl‐sulfate polyacrylamide gel electrophoresis (SDS‐PAGE). The proteins were transferred onto a Polyvinylidene fluoride (PVDF) membrane and blocked with either 5% skim milk or 5% BSA at room temperature to minimize nonspecific binding.

Following washing with Tris‐Buffered Saline with Tween‐20 (TBST) (TBS + 0.1% Tween‐20), the membranes were incubated overnight at 4°C with primary antibodies targeting the proteins of interest. After additional TBST washes, the membranes reacted with horseradish peroxidase (HRP)‐conjugated secondary antibodies (Genetex, Irvine, CA, USA) for an hour at room temperature. Finally, the signals were detected with enhanced chemiluminescence (ECL) reagent and acquired by the luminescence imaging system ImageQuant™ LAS‐4000 (G.E., MA, USA). Band intensities were quantified using ImageJ, with β‐actin serving as the internal CTRL. All the protein expressions were first normalized with their corresponding β‐actin signal, and the relative protein expressions were obtained by dividing the expressions in treated cells by those of the CTRL group. The information on antibodies is listed in Table S1.

### 2.9 In vivo therapeutic efficacy

5‐week‐old male BALB/c mice and BALB/c Nude mice were purchased from the National Laboratory Animal Center, Taiwan (IACUC No. 1101012, 1111010). 1 × 10^6^ CT26 cells were inoculated into the right flank of BALB/c mice, while 2 × 10^6^ HCT116 cells were inoculated into the right flank of BALB/c Nude mice. Two cell lines were inoculated with different concentrations based on their origins. As mentioned, CT26 is a murine‐origin cell line, and HCT116 is a human‐origin cell line. According to our previous experiences, tumors will grow better with these specific concentrations during inoculation. Treatments began when tumor volumes reached 60–80 mm^3^. Tumor‐bearing mice were divided into four groups, including CTRL, CUR, MET, and combination treatment (COMB). CUR was dissolved in DMSO and then diluted with ddH_2_O to a final concentration of 20 mg/kg (5% DMSO). A 200 mg/kg MET solution was prepared with ddH_2_O. Both CUR and MET solutions were administered intraperitoneally for 14 days, with tumor volume and body weight monitored every 3 days until the end of the experiment. Tumor volume was calculated using the formula: Tumor volume (mm^3^) = 0.5 × length × width^2^.

### 2.10 Hematoxylin and eosin staining

Paraffin‐embedded tumor sections were deparaffinized using xylene and rehydrated with graded ethanol. Following PBS washes, tumor sections were immersed in hematoxylin and eosin to stain the cell nuclei and cytoplasm. Then, tumor sections were dehydrated with gradient ethanol and xylene. Finally, tumor sections were covered by a mounting medium and sealed. The H&E staining images were captured using an Olympus BX‐61 equipped with a 20× objective.

### 2.11 Statistical analysis

Data were analyzed using GraphPad Prism 9 software version 10.0.3 (GraphPad Software Inc.; San Diego, CA, USA). The Analysis of variance (ANOVA) was used for comparisons. The data was presented as mean with error bars indicating standard error of the mean (SEM). A value of *p* < 0.05 indicates a statistical significance between groups.

## 3 RESULTS

### 3.1 CUR combined with MET downregulates DMT1 level and suppresses cell growth by downregulating the pAKT/mTOR/cyclin D1 signaling pathway

**FIGURE 1:**
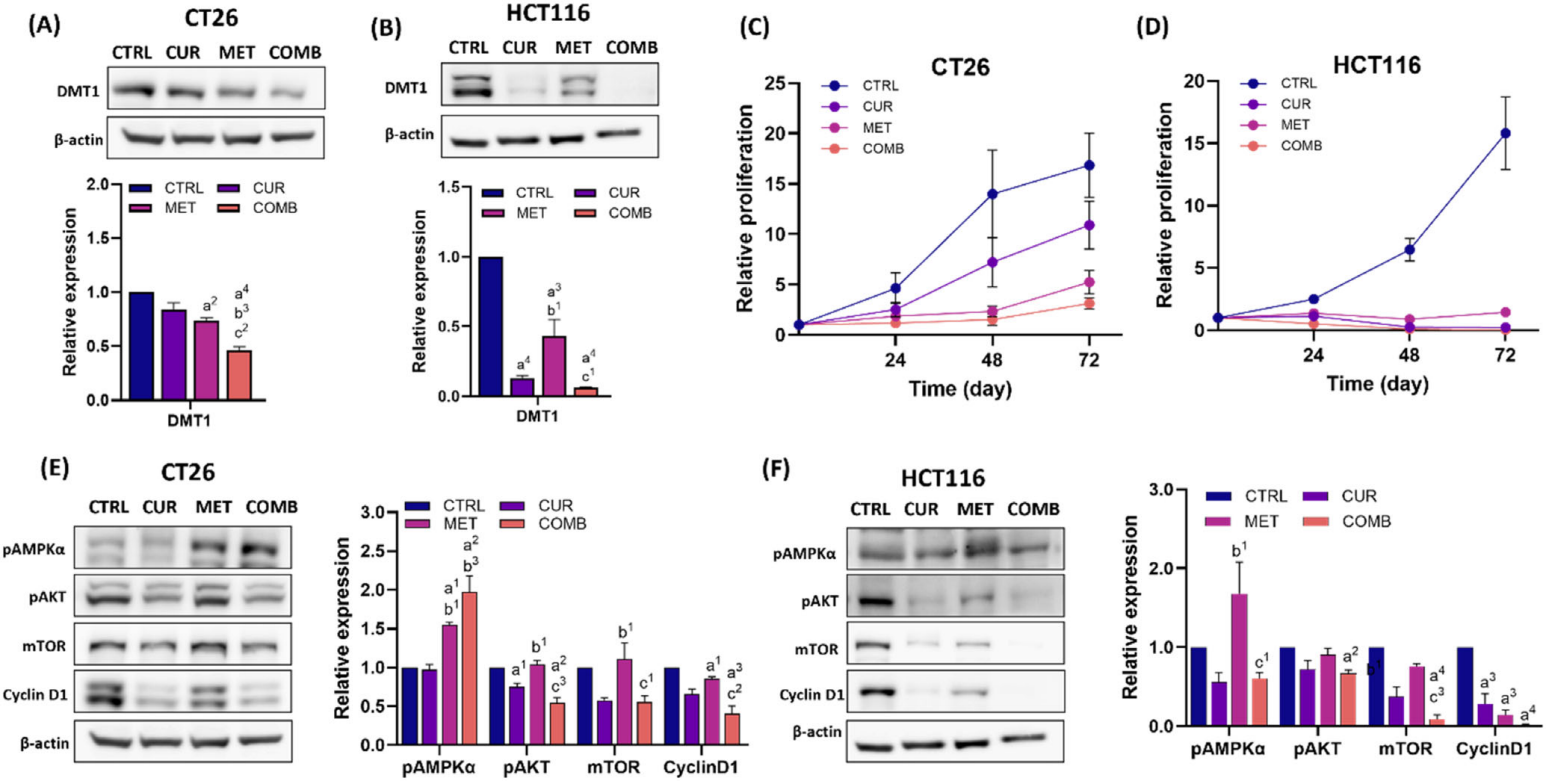
Combination treatment suppresses cell proliferation by downregulating pAKT/mTOR/Cyclin D1 pathways. (A, B) Representative Western blotting images and quantitative results of DMT1 in CT26 and HCT116 cells. (C, D) Cell proliferation monitoring in CT26 and HCT116 cells after receiving different treatments. The cell numbers obtained at the designated time points were normalized to the seeded cell number (0 h). (E, F) Representative Western blotting images and quantitative results of pAMPKα, pAKT, mTOR, and Cyclin D1 in CT26 and HCT116 cells. β‐Actin was used as an internal control, and all the protein expression levels were normalized to that of the CTRL group. Results of three biological replicates, with error bars indicating SEM. (C, D) Two‐way ANOVA was used. (a) Comparison between CTRL and CUR; (b) comparison between CTRL and MET; (c) comparison between CTRL and COMB; (1) *p* < 0.05; (2) *p* < 0.01; (3) *p* < 0.001; (4) *p* < 0.0001. (A–B, E–F) One‐way ANOVA was used. (a) Compared to CTRL; (b) compared to CUR; (c) compared to COMB. (1) *p* < 0.05; (2) *p* < 0.01; (3) *p* < 0.001; (4) *p* < 0.0001.

Both curcumin (CUR group) and metformin (MET group) downregulated the expression of DMT1 in CT26 and HCT116 cells, with a more pronounced effect observed when combined CUR with MET (COMB group) (Figure 1A,B). Notably, CUR caused more significant inhibition of DMT1 in HCT116 cells compared to CT26 cells.

The IC50 concentrations of CUR and MET were determined by the MTT assay. In CT26 cells, the IC50 values for CUR and MET were approximately 15 μM and 10 mM, respectively. In HCT116 cells, the IC50 values were about 50 μM and 40 mM, respectively (Figure S1). We then treated cells with CUR and MET simultaneously at their respective IC50 concentrations to study how the anti‐proliferative effect would be affected. Both CUR and MET effectively inhibited cell growth in both cell lines during the initial 48 h, with a stronger effect observed in HCT116 cells (Figure 1C,D). Notably, CT26 cells treated with CUR alone showed repopulation starting from the 48‐h time point, whereas this phenomenon was absent in HCT116 cells and the combination group, indicating a more sustained growth inhibition with the COMB.

MET is a well‐known AMPK activator, which was confirmed in our study. As shown in Figure 1E,F, the protein expression changes caused by treatments in CT26 and HCT116 cells. In CT26 cells, the COMB group had the highest pAMPKα expression accompanied by lower pAkt and mTOR expressions compared to other groups. CUR alone decreased pAkt and mTOR expression but did not alter pAMPKα level. Both CUR and COMB groups showed reduced Cyclin D1 expression. With regard to HCT116 cells, MET increased pAMPKα expression, but this increment was not further enhanced by the COMB. However, the COMB group also showed suppression of both pAkt and mTOR expressions, and all treatments significantly reduced Cyclin D1 levels.

In summary, MET or CUR effectively suppressed cell proliferation in both cell lines, and this anti‐proliferative effect was further augmented when the two agents were combined together. Considering the heterogeneity of the two CRC cells on pAMPK expression, this augmenting anti‐proliferative effect may be attributed, at least in part, to the inhibition of the pAkt/mTOR/Cyclin D1 pathway.

### 3.2 COMB enhances the generation of ROS by exerting distinct regulatory influences on NRF2/KEAP1 in CT26 and HCT116 cells

**FIGURE 2:**
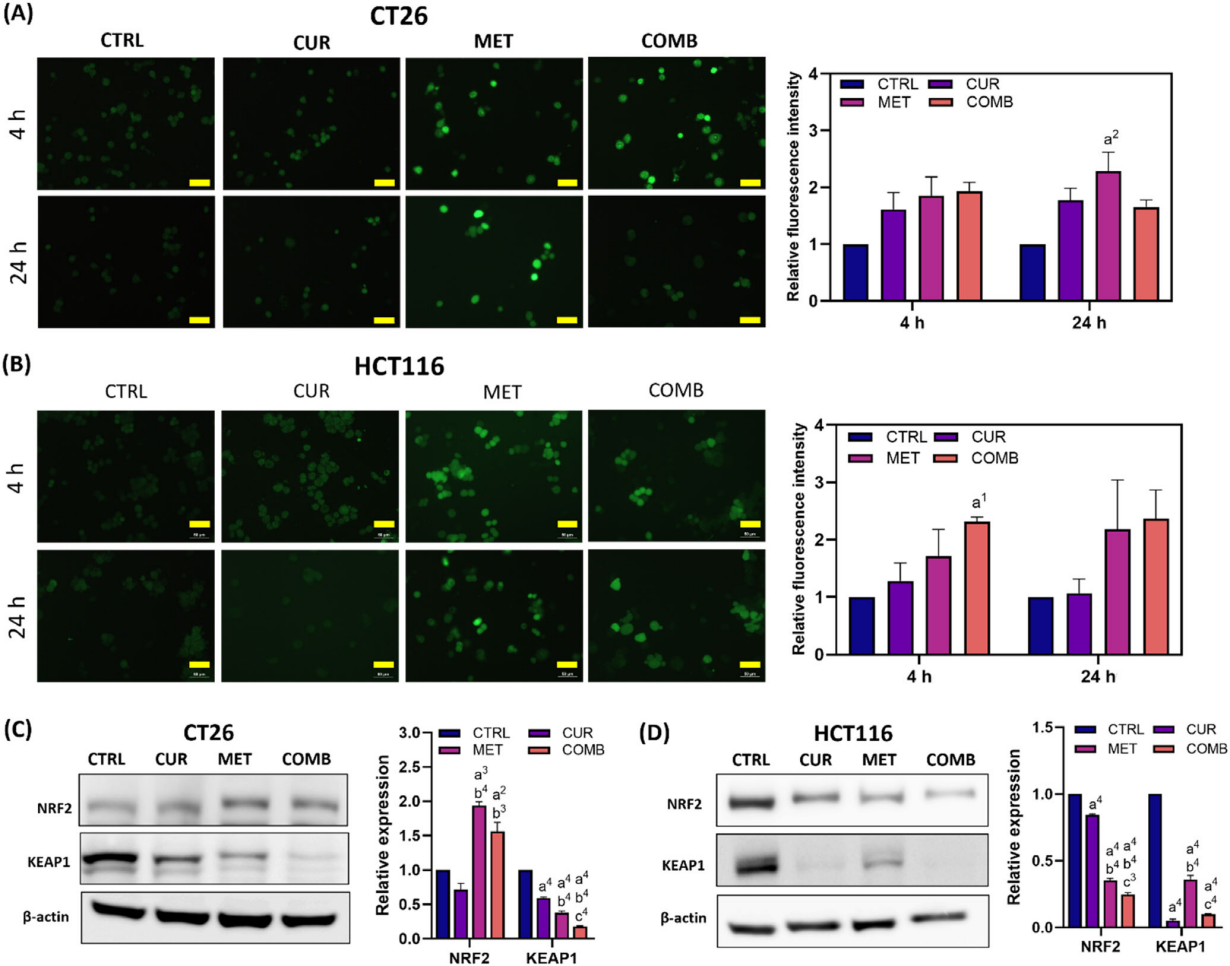
Combination treatment induces substantial ROS generation and inactivates KEAP1 expression. The representative DCFH‐DA staining images and quantitative results of (A) CT26 and (B) HCT116 cells receiving different treatments were acquired at the indicated time points. Quantification results were normalized to those of the CTRL group at each time point. The yellow line represents the scale bar = 50 μm. Representative Western blotting images of NRF2 and KEAP1 and the quantitative results in (C) CT26 and (D) HCT116 cells. β‐Actin served as an internal control, and protein expression levels were normalized to the CTRL group. Results of three biological replicates, with error bars indicating SEM. One‐way ANOVA was used. (a) compared to CTRL; (b) compared to CUR; (c) compared to COMB. (1) *p* < 0.05; (2) *p* < 0.01; (3) *p* < 0.001; (4) *p* < 0.0001.

We evaluated intracellular ROS levels in CT26 and HCT116 cells subjected to various treatments using DCFH‐DA staining. Representative images and quantitative results of DCFH‐DA staining in CT26 cells are presented in Figure 2A. Elevated ROS signals were detected in all the treated groups at the 4‐h time point, persisting until the 24‐h time point. Similar trends were observed in HCT116 (Figure 2B), with MET exhibiting more substantial ROS production than CUR. Moreover, simultaneous treatment with both compounds resulted in augmented ROS generation. Interestingly, CUR induced more pronounced ROS production in CT26 cells compared to HCT116 cells, with CUR‐induced ROS levels returning to baseline by the 24‐h time point. In summary, both CUR and MET can induce ROS generation, with MET appearing more effective than CUR.

Under normal circumstances, KEAP1 regulates the activity and nuclear translocation of NRF2 by being part of the E3 ubiquitin ligase complex. Oxidative stress inactivates KEAP1, leading to NRF2 nuclear translocation and elevated antioxidant gene expression to regulate redox balance. Figure 2C,D demonstrate the changes in NRF2 and KEAP1 expression in CT26 and HCT116 cells after being treated for 24 h. Although CUR reduced KEAP1 expression more effectively in HCT116 cells than in CT26 cells, all treatments resulted in a significant KEAP1 reduction in both cell lines, with the combination therapy showing the greatest inhibition. Interestingly, the MET and COMB groups exerted distinct effects on NRF2 expression in these two cell lines. Following MET and combination therapy, CT26 cells showed a significant increase, while HCT116 cells displayed a reduction in NRF2 expression. However, CUR exhibited similar effects on NRF2 expression in both cell lines. These findings implicate that combined treatment may reduce KEAP1 expression and subsequently promote Nrf2 nuclear translocation and antioxidant gene expression in both cell lines.

### 3.3 COMB elevates Bax/Bcl‐2 ratio and leads to significant cell death

With the COMB group demonstrating significant cell death and ROS production in both cell lines, our focus shifted toward understanding the potential relationship between elevated ROS levels and cell death, along with elucidating underlying mechanisms. We assessed changes in apoptosis‐related proteins using Western blotting analysis.

**FIGURE 3:**
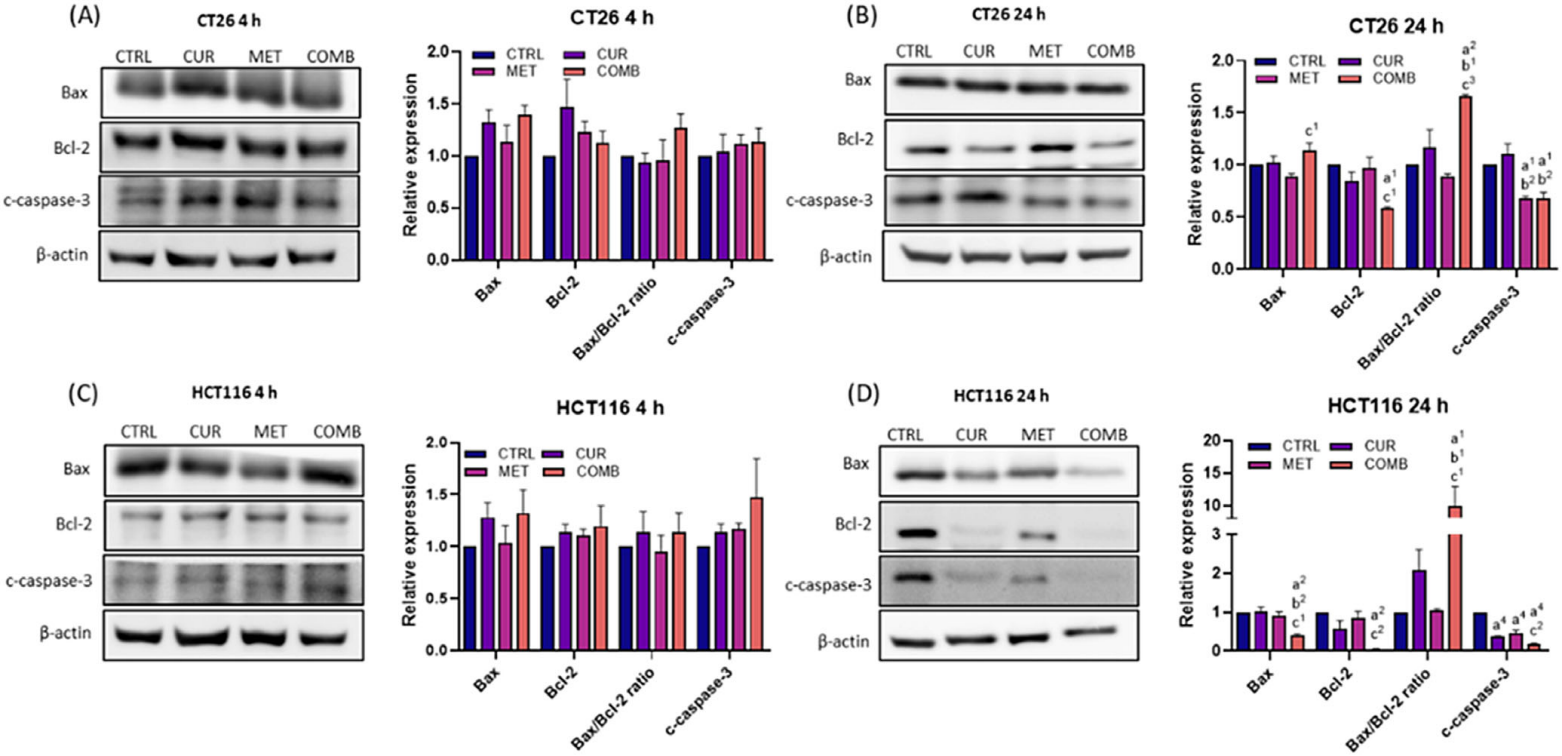
Curcumin combined with metformin increases the Bax/Bcl‐2 ratio in CRC cells. The representative Western blotting images of apoptosis‐associated proteins and the corresponding quantification results obtained from CT26 cells receiving various treatments at (A) 4‐ and (B) 24‐h time points. The representative Western blotting images of apoptosis‐associated proteins and the corresponding quantification results obtained from HCT116 cells receiving various treatments at (C) 4‐ and (D) 24‐h time points. β‐Actin served as an internal control, and protein expression levels were normalized to the CTRL group. Results of three biological replicates, with error bars indicating SEM. One‐way ANOVA was used. (a) compared to CTRL; (b) compared to CUR; (c) compared to COMB. (1) *p* < 0.05; (2) *p* < 0.01; (3) *p* < 0.001; (4) *p* < 0.0001.

In CT26 cells, slight increases in Bax, Bax/Bcl‐2 ratio and cleaved caspase‐3 were detected in both CUR and COMB groups at the 4‐h time point as compared to the CTRL group, but there was no significant difference (Figure 3A). By the 24‐h time point, comparable Bax expressions were noted across all groups. At the same time, a significant reduction in Bcl‐2 was observed, specifically in the COMB group (Figure 3B). Consequently, the Bax/Bcl‐2 ratio remained highest in the COMB group compared to other treatment groups. Surprisingly, the MET and COMB groups showed downregulation of cleaved caspase‐3 compared to the CTRL group.

Similar patterns were observed in HCT116 cells (Figure 3C,D), with differences appearing only at the 24‐h time point. Here, the COMB group exhibited the lowest levels of Bax, Bcl‐2, and cleaved caspase‐3 compared to other groups, maintaining the highest Bax/Bcl‐2 ratio. A substantial increase in the Bax/Bcl‐2 ratio indicates potential mitochondria damage and apoptosis initiation caused by the COMB. However, evidence of apoptosis remains inconclusive, as cleaved caspase‐3 levels were diminished in all treated groups at the 24‐h time point, particularly noticeable in the MET and COMB groups in both cell lines, although cleaved caspase‐3 was slightly increased in MET and COMB groups at 4‐h time point.

### 3.4 CUR enhances autophagy induced by MET treatment in CT26 and HCT116 cells

Despite the significant suppression of the anti‐apoptotic protein Bcl‐2 after treatment, no notable change in cleaved caspase‐3 was observed, precluding a conclusive determination that the observed cell death resulted from apoptosis. To explore the underlying cause of cell death, we shifted our focus to changes in autophagic‐related proteins in both cell lines. Additionally, ROS is known to induce autophagic cell death, a self‐protective mechanism, by regulating mTOR and the NRF2/KEAP1 pathway. The protein p62 inhibits autophagy by interacting with NRF2‐KEAP1 complexes, while LC3 serves as a marker for autophagosome formation. The cytosolic form of LC3, known as LC3‐I, is conjugated with phosphatidylethanolamine, converting it into the membrane‐bound form, LC3‐II. LC3‐II is then recruited for the formation of autophagosomes, a critical component of autophagy.

**FIGURE 4:**
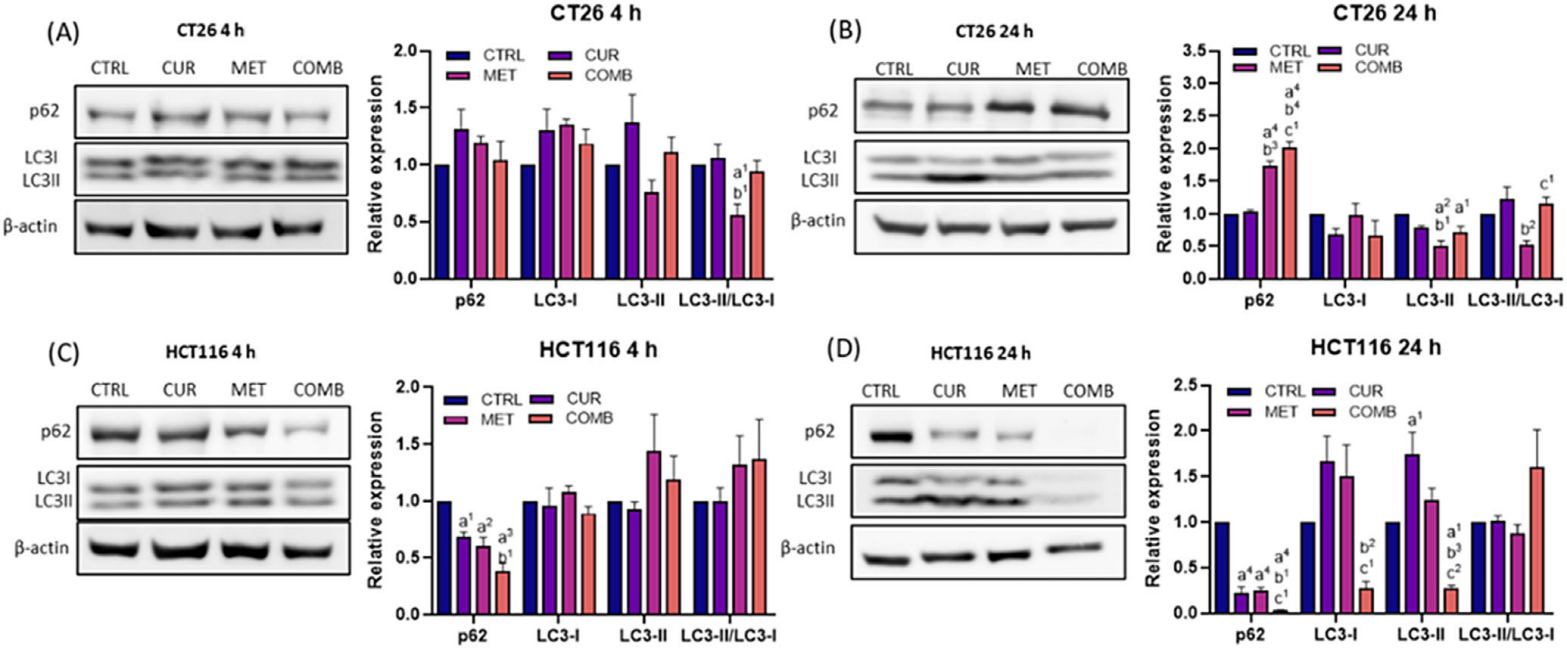
Curcumin enhances metformin‐mediated autophagy. The representative Western blotting images of autophagy‐associated proteins and the corresponding quantification results obtained from CT26 cells receiving various treatments at (A) 4‐ and (B) 24‐h time points. The representative Western blotting images of apoptosis‐associated proteins and the corresponding quantification results obtained from HCT116 cells receiving various treatments at (C) 4‐ and (D) 24‐h time points. β‐Actin served as an internal control, and protein expression levels were normalized to the CTRL group. Results of three biological replicates, with error bars indicating SEM. One‐way ANOVA was used. (a) compared to CTRL; (b) compared to CUR; (c) compared to COMB. (1) *p* < 0.05; (2) *p* < 0.01; (3) *p* < 0.001; (4) *p* < 0.0001.

Figure 4A,B show autophagy‐related protein expressions in CT26 cells at 4‐ and 24‐h post‐treatment. At 4‐h post‐treatment, both CUR and MET groups displayed slight increases in p62, while all treatment groups exhibited elevated levels of LC3‐I. Simultaneously, the MET group showed a decrease in LC3‐II, whereas the CUR and COMB groups displayed a slight increase. By the 24‐h time point, p62 levels returned to baseline in the CUR group but increased significantly in the MET and COMB groups. Both the CUR and COMB groups showed comparable levels of LC3‐I and LC3‐II, while the MET group had higher LC3‐I and lower LC3‐II expressions. Compared to the CTRL group, the LC3‐II/LC3‐I ratio slightly increased in the CUR and COMB groups but declined in the MET group.

Figure 4C,D depict autophagy‐related protein expressions in HCT116 cells. In contrast to CT26 cells, all treatments led to a significant reduction in p62 at 4‐h post‐treatment, further decreasing at 24 h. Notably, the COMB group had the lowest p62 levels. The MET and COMB groups showed higher LC3‐II/LC3‐I ratios at 4‐h post‐treatment compared to the CTRL and CUR groups. Importantly, only COMB increased the ratio at 24‐h post‐treatment, indicating that MET‐mediated autophagy can be sustained when administered concurrently with CUR.

Based on the results of LC3‐II/LC3‐I ratio at 24‐h post‐treatment, MET alone inhibits autophagy in CT26 cells and slightly inhibits it in HCT116 cells. The COMB group had a higher ratio than the MET group in both cell lines, suggesting that CUR has the potential to enhance MET‐regulated autophagy in CT26 and HCT116 cells via p62‐independent manner. It may be due to downregulating KEAP1 expression and promoting Nrf2 nuclear translocation in both cell lines, resulting in an elevated LC3‐II/LC3‐I ratio.

### 3.5 CUR combined with MET induces lipid peroxidation and promotes ferroptosis in CT26 and HCT116 cells

**FIGURE 5:**
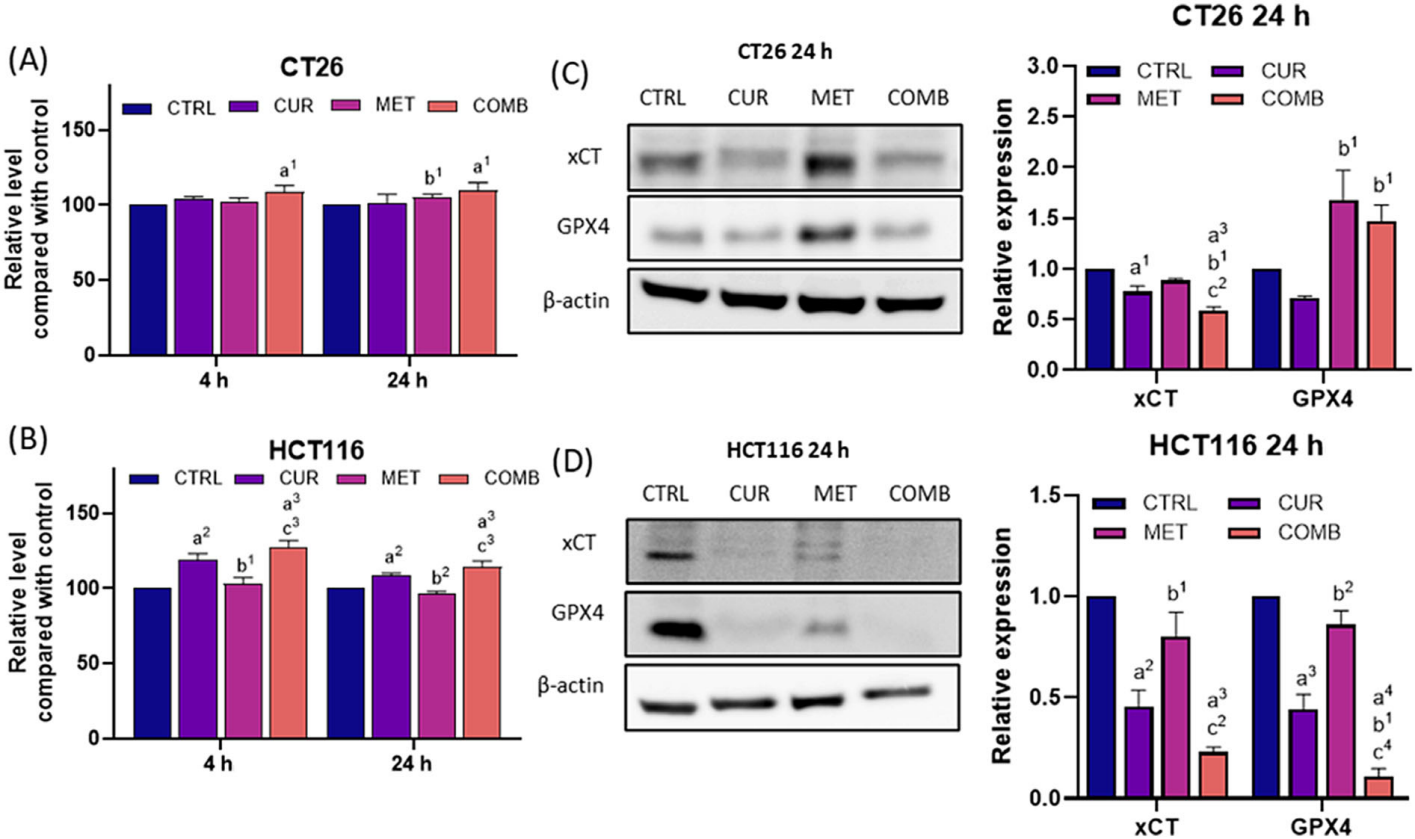
Curcumin combined with metformin induces lipid peroxidation and downregulates xCT‐GPX4 axis in CRC. (A, B) The relative lipid peroxidation levels of different groups at 4‐ and 24‐h time points in CT26 and HCT116 cells. (C, D) The representative Western blotting images and the corresponding quantification results of xCT and GPX4 obtained from CT26 and HCT116 cells received various treatments. β‐Actin served as an internal control, and protein expression levels were normalized to the CTRL group. Results of three biological replicates, with error bars indicating SEM. One‐way ANOVA was used. (a) compared to CTRL; (b) compared to CUR; © compared to COMB. (1) *p* < 0.05; (2) *p* < 0.01; (3) *p* < 0.001; (4) *p* < 0.0001.

In addition to inducing apoptosis and autophagy, excessive ROS can lead to various adverse modifications, including the oxidation of polyunsaturated fatty acids (PUFAs). PUFAs, crucial components of cellular membranes, are highly susceptible to ROS, resulting in lipid peroxidation‐associated ferroptosis. Figure 5A,B demonstrated the changes in the levels of lipid peroxidation in CT26 and HCT116 cells, showing an increase in COMB groups at the 24‐h time point.

In response to ROS‐induced lipid peroxidation, the xCT‐GPX4 axis plays a crucial role in neutralizing lipid peroxides. Inactivation of xCT‐GPX4 typically leads to cellular ferroptosis. Figure 5C,D showed that MET did not significantly alter the expression of xCT, while COMB inhibited xCT in both cell lines. In contrast, GPX4 changes varied between the two cell lines. CT26 cells showed increased GPX4 in COMB group, whereas HCT116 cells exhibited a significant reduction in GPX4 in COMB groups.

Considering the heterogeneity of the two CRC cells on GPX4 expression, our research indicates an increase in lipid peroxidation with combined treatment, alongside a downregulation of the xCT expression compared to MET treatment alone when combined with CUR. These findings suggest that the combined treatment leads to ROS‐associated ferroptosis due to lipid peroxidation‐downregulated xCT expression.

### 3.6 MET enhances the CUR‐mediated NF‐κB suppression in CT26 and HCT116 cells

**FIGURE 6:**
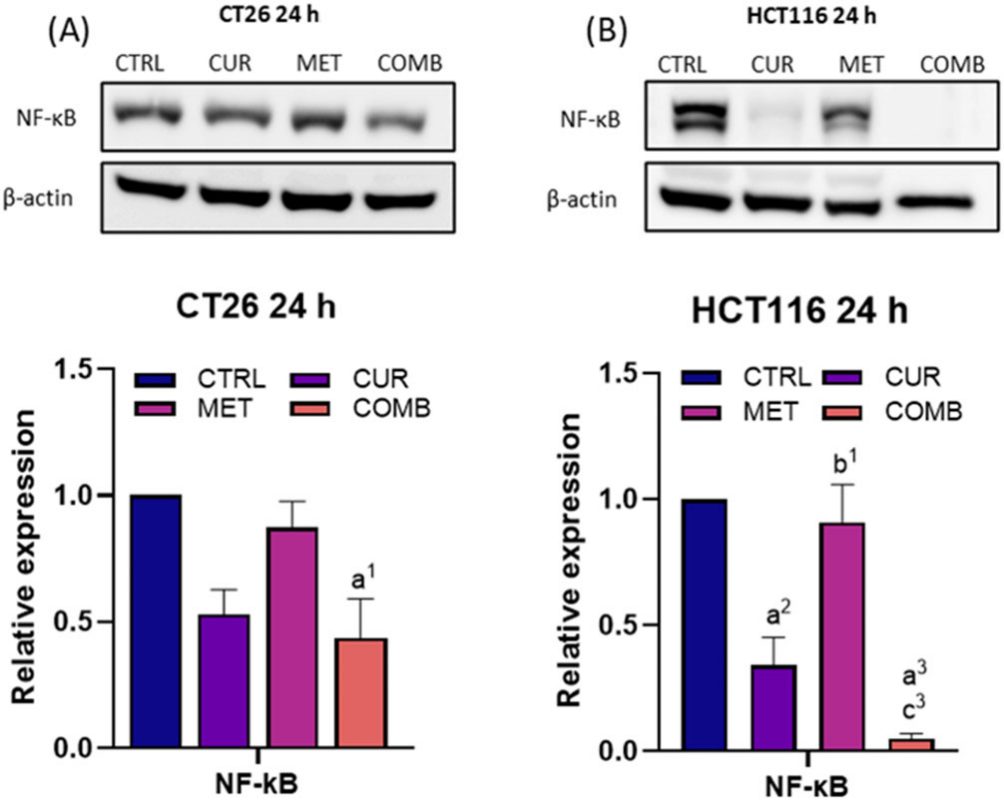
Combined curcumin with metformin decreases NF‐κB levels in CRC cells. The Western blotting images and the quantification result of NF‐κB in (A) CT26 and (B) HCT116 cells at 24‐h time points. β‐Actin served as an internal control, and protein expression levels were normalized to the CTRL group. Results of three biological replicates, with error bars indicating SEM. One‐way ANOVA was used. (a) compared to CTRL; (b) compared to CUR; (c) compared to COMB. (1) *p* < 0.05; (2) *p* < 0.01; (3) *p* < 0.001; (4) *p* < 0.0001.

NF‐κB serves a crucial role in cancer progression by acting as a transcription factor and regulating the expression of genes involved in cell proliferation and cell death. Figure 6 illustrates that both CT26 and HCT116 cells exhibited similar responses to all treatments. Notably, the COMB group demonstrated the most significant reduction in NF‐κB expression, followed by the CUR group. CUR showed stronger inhibition of NF‐κB compared to MET alone. Moreover, the COMB group induced a more pronounced downregulation of NF‐κB in HCT116 cells than in CT26 cells. These findings suggest that combining CUR with MET has a greater reduction in NF‐κB expression that is primarily regulated by CUR, resulting in the inhibition of cell proliferation.

### 3.7 COMB effectively diminishes the migratory ability of CT26 and HCT116 cells

**FIGURE 7:**
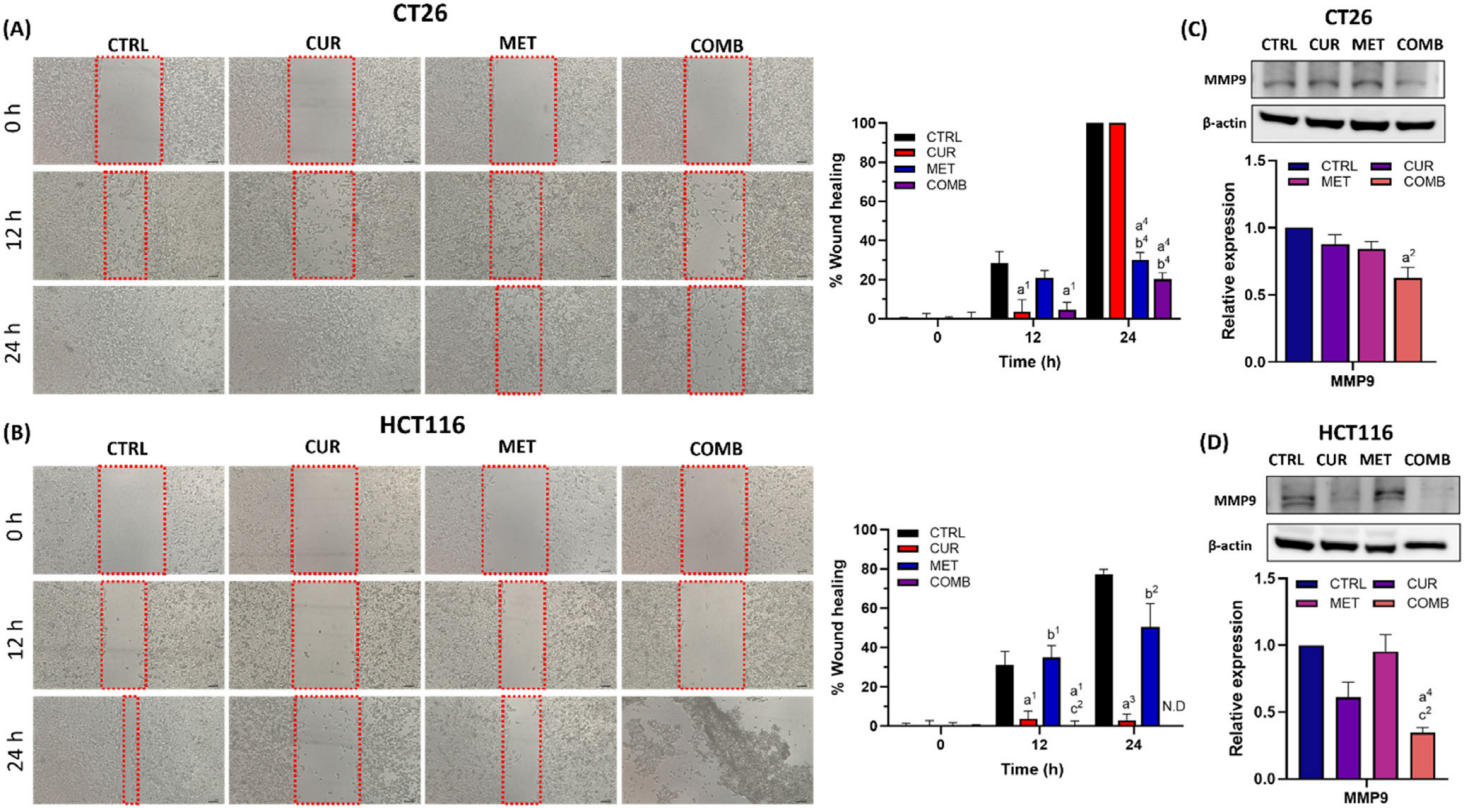
Combination treatment exhibits the most significant inhibition on cell migration in CRC cells. Representative cell migration images and the corresponding quantification results of the percentage of wound healing of (A) CT26 and (B) HCT116 cells. Representative Western blotting images and quantitative results of MMP9 of (C) CT26 and (D) HCT116 cells. β‐Actin served as an internal control, and protein expression levels were normalized to the CTRL group. Results of three biological replicates, with error bars indicating SEM. One‐way ANOVA was used. (a) compared to CTRL; (b) compared to CUR; (c) compared to COMB. (1) *p* < 0.05; (2) *p* < 0.01; (3) *p* < 0.001; (4) *p* < 0.0001.

Distal metastasis is a primary cause of cancer‐related mortality, notably in CRC patients. Accordingly, the migration capacity of both cell lines was assessed by the wound healing assay following various treatments. Figure 7A,B show the images of CT26 and HCT116 cells at designated time points post‐treatment, alongside the corresponding percentages of wound healing relative to the first time point. At the 12‐h time point, wound healing percentages of CT26 cells were 28.4%, 3.7%, 20.9%, and 4.5% for CTRL, CUR, MET, and COMB groups, respectively. The wound closure percentages increased to 100%, 100%, 30.2%, and 20.1% at the 24‐h time point. Similarly, wound healing percentages of HCT116 cells were 31.2%, 3.5%, 34.9%, and 0% at 12‐h and 77.4%, 2.8%, and 50.4% at the 24‐h time point for CTRL, CUR, and MET groups, respectively. Notably, in HCT116 cells, the COMB group at 24 h. displayed significant cell death and detachment, making migration results indiscernible. Quantification results at the 12‐ and 24‐h time points revealed similar migratory behavior in both cell lines, with the MET group showing no significant difference from the CTRL group in repressing cell migration. In contrast, the CUR group demonstrated a robust anti‐migration effect, and the inhibition of cell migration in the COMB group was even greater than in both the MET and CUR groups. These results emphasize that the observed inhibition of cell migration in the COMB group was predominantly due to CUR.

Next, we determined the level of MMP9, a migration‐associated protein, through Western blotting to validate wound healing results. Figure 7C,D display representative Western blotting images and quantified results of MMP9 in CT26 and HCT116 cells at the 24‐h time point. Consistent with the wound healing assay results, the COMB groups exhibited the lowest MMP9 levels in both cell lines, indicating that COMB could suppress cellular migration by decreasing MMP9 expression.

### 3.8 The combination of CUR and MET significantly enhances anti‐tumor abilities compared to individual treatments in CRC‐bearing mice

**FIGURE 8:**
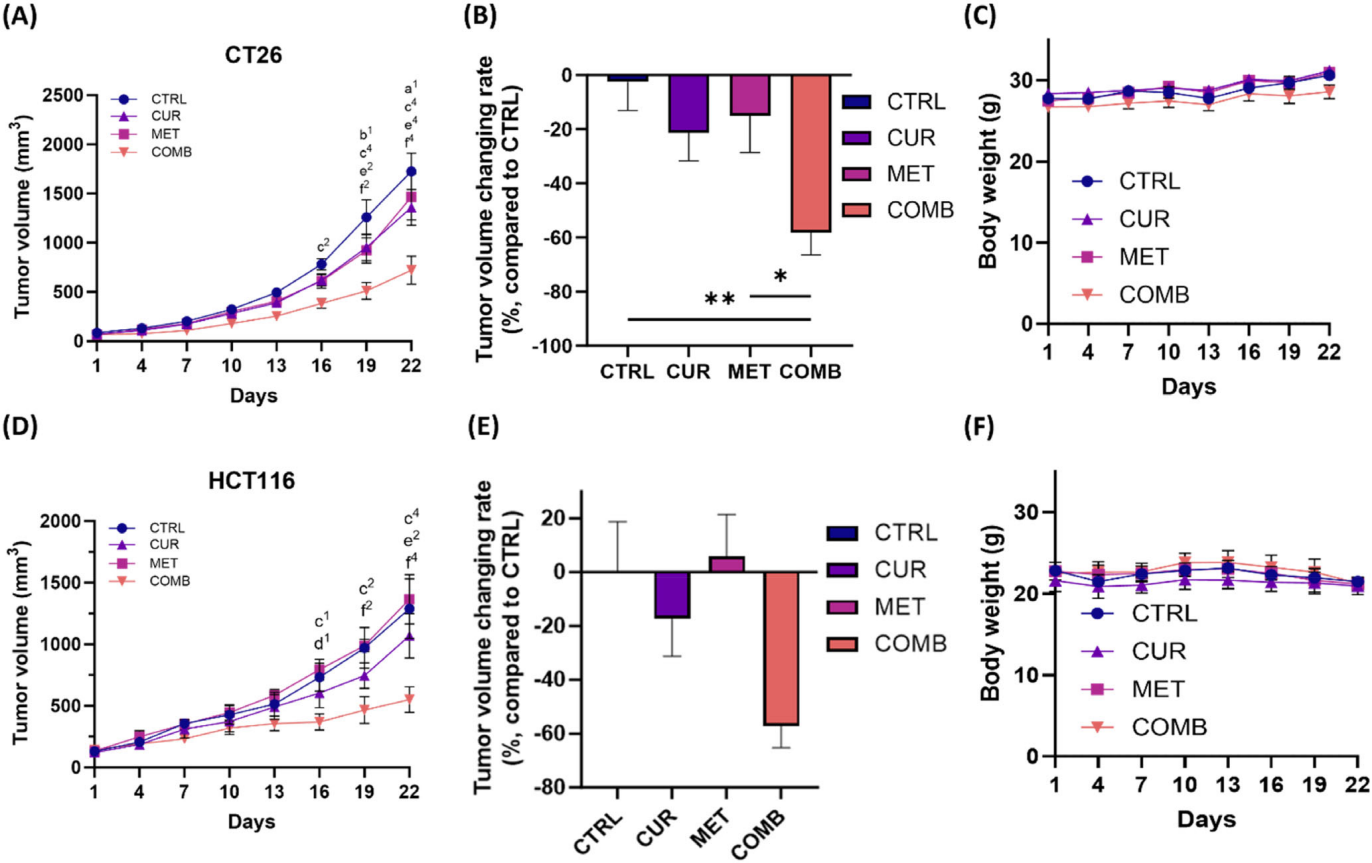
Combined treatment demonstrates anti‐tumor efficacy in vivo in CT26 and HCT116 tumor‐bearing mice. (A) Tumor growth curves of each group. (B) Tumor inhibition rate calculated from tumor growth curve results on day 22. (C) Changes of body weight in each group in CT26 tumor‐bearing mice (*n* = 7 per group). (D) Tumor growth curves of each group. (E) Tumor inhibition rate calculated from tumor growth curve results on day 22. (F) Changes of body weight in each group in HCT116 tumor‐bearing mice (*n* = 3 per group). (A) Comparison between CTRL and CUR; (B) comparison between CTRL and MET; (C) comparison between CTRL and COMB; (D) comparison between CUR and MET; (E) comparison between CUR and COMB; (1) *p* < 0.05; (2) *p* < 0.01; (3) *p* < 0.0001. (A, D) Two‐way ANOVA was used. (a) Comparison between CTRL and CUR; (b) comparison between CTRL and MET; (c) comparison between CTRL and COMB; (d) comparison between CUR and MET; (e) comparison between CUR and COMB; (1) *p* < 0.05; 2, *p* < 0.01; (3) *p* < 0.001; (4) *p* < 0.0001. (B) One‐way ANOVA was used. *p < 0.05; **p < 0.01.

We established CT26 and HCT116 tumor‐bearing mouse models to evaluate the impact of COMB on tumor growth. Figure 8A–C and D–F illustrate tumor growth, tumor inhibition, and body weight changes in CT26 and HCT116 tumor‐bearing mice subjected to different treatments, respectively. Tumor growth curves revealed that CUR and MET groups displayed 15%–20% tumor inhibition in the CT26 model, and MET did not slow down tumor progression in the HCT116 model. COMB group exhibits the most substantial tumor inhibition when compared with other groups. Notably, the COMB group demonstrated nearly 60% tumor inhibition compared to the CTRL group in both models on Day 22 post‐treatment. Importantly, none of these treatments led to general toxicity; there was no weight loss observed during the study period, and organ damage was undetectable at the end of the experiment (Figure 8C,F, and Figure S2).

## 4 DISCUSSION

Both CUR and MET have demonstrated anti‐cancer capacity by modulating ROS production, which subsequently initiates different cell death mechanisms, including apoptosis, autophagy, and ferroptosis. However, the effects of combining these two drugs on ROS production and subsequent cell death remain unclear. Therefore, we investigated the therapeutic effects and mechanisms of the combination of CUR and MET in both cell culture and tumor‐bearing mice models.

Previous studies have explored the correlation between iron levels and the development of various cancers, including CRC.^21^, ^22^ These correlations may be partly associated with the essential role of iron metabolism in oxygen transport, DNA synthesis, and electron transport.^23^ Transferrin transports ferric ions (Fe^3+^) from the circulation into endosomes of hepatocytes and intestinal epithelium, where they are reduced to ferrous ions (Fe^2+^) by STEAP3 and then transported to the cytosol by DMT1.^24^ DMT1‐based iron uptake has been shown to promote CRC tumorigenesis.^6^ Iron serves as a cofactor for enzymes involved in essential biochemical processes. Downregulation of DMT1 could disrupt the balance of Fe^2+^ and other essential metal ions, such as Zn^2+^, Mn^2+^, Cu^2+^, and Ni^2+^ levels, inducing cellular stress and impairing cellular functions.^25^ Our results further demonstrate that COMB with CUR and MET effectively downregulates DMT1 expression in both CT26 and HCT116 cells (Figure 1A,B), correlating with the anti‐proliferation effect (Figure 1C,D). Aberrant activation of PI3K/AKT, MAPK, and NF‐κB pathways is known to lead to uncontrollable cell growth.^26^, ^27^ Xiao et al. proved that MET can downregulate the IN‐HBA/TGFβ/PI3K/AKT/CyclinD1 pathway in CRC cells.^28^ Further, our data suggest that the anti‐proliferation in CRC cells caused by combining CUR with MET is partially related to the downregulated pAKT/mTOR/Cyclin D1 signaling pathway (Figure 1E,F), likely due to the dysfunction of the iron transporter DMT1.

ROS plays a pivotal role in inducing cellular stress and DNA damage, thereby activating programmed cell death pathways like apoptosis, autophagy, and ferroptosis.^29^ MET is a widely recognized ROS inducer due to its mitochondrial complex I inhibition property.^30^, ^31^ Liu et al.^16^ demonstrated that CUR elevates ROS levels in HCT116 cells using the DCFH‐DA staining assay. CUR can also stimulate ROS production,^32^, ^33^ which is largely dependent on dosage and time points.^34^, ^35^ Similarly, an elevating ROS was also observed in the two CRCs after treating CUR or MET (Figure 2A,B). In particular, we further noted that this effect was enhanced by a combination of treatment with CUR and MET.

The KEAP1‐NRF2 axis plays a central role in the oxidative stress response.^36^ ROS modifies the cysteine residues of KEAP1, allowing NRF2 to escape from KEAP1, and the activated NRF2 then translocates to the nucleus to increase the transcription of antioxidant genes. Although ROS production induced by CUR, MET, or a combined strategy is followed by KEAP1 inactivation, the response of NRF2 differs between CT26 and HCT116 cells after combined treatment (Figure 3C,D). In CT26 cells treated with CUR and MET, ROS‐induced KEAP1 downregulation increases NRF2 levels. However, in HCT116 cells treated with CUR and MET, both KEAP1 and NRF2 levels were downregulated after treatments. In cancer cells, KEAP1‐independent pathways, such as AMPK/mTOR^37^ and AKT,^38^ might contribute to NRF2 regulation. Activation of AKT and AMPK can inactivate GSK3, a secondary mediator of NRF2 degradation, thereby facilitating the nuclear translocation of NRF2.^39^ CUR and MET exhibit different regulatory effects on pAKT and pAMPKα in monotherapy,^40^ implicating the possible response to these proteins varies in the different cells after combined treatment. In alignment with these findings, we observed an upregulation of NRF2 in CT26 cells but a downregulation in HCT116 cells in COMB groups.

Excess ROS generation typically triggers cellular apoptosis.^41^ CUR is recognized for its ability to induce Bax/Bcl2‐involved apoptosis in cancer cells,^16^, ^42^ as evidenced by the elevated levels of Bax, Bax/Bcl2 and cleaved caspase‐3 in both CT26 and HCT116 cells treated with CUR at the 4‐h time point in our study (Figure 3A,C). By 24‐h post‐treatment, the levels of Bcl‐2 and caspase‐3 in both cell lines treated with CUR had significantly decreased (Figure 3B,D). Of note, these effects were enhanced by combined treatment at 4‐ and 24‐h post‐treatment. This suggests that combined treatment‐upregulated Bax occurs early while combined treatment‐downregulated Bcl‐2 happens later. However, evidence of apoptosis remains inconclusive, as cleaved caspase‐3 levels were diminished in CUR and combined treatment groups at the 24‐h time points. Since the increase in the Bax/Bcl2 ratio also induces mitochondria damage in cancer cells, leading to autophagic cell death via a caspase‐3‐independent manner.^43^, ^44^ According to these findings, we speculated that a combined treatment‐enhanced Bax/Bcl‐2 ratio at the 24‐h time point in both cell lines may induce potential mitochondria damage‐related non‐apoptotic cell death via caspase‐3‐independent manner.

In addition to cell death, excessive ROS can initiate autophagy to remove damaged organelles.^45^ Autophagy serves as a self‐protective mechanism to clear oxidized cellular components and regulate cellular ROS levels. However, severe damage can still lead to cell death.^46^, ^47^, ^48^ The interplay between cell apoptosis and autophagy involves various proteins, including death receptors and members of the Bcl‐2 family. Downregulation of Bcl‐2 and Bcl‐xL can promote autophagy.^48^, ^49^ Roncucci et al.^50^ confirmed that MET increases oxidative stress and triggers autophagy, aligning with our findings in both cell lines (Figure 4B,D). Although our results reveal distinct trends in p62 expression across two cell lines, upon downregulating KEAP1 expression in both cell lines, KEAP1 typically binds to p62 and sequesters it into autophagosomes. Additionally, the nuclear translocation of NRF2 triggers p62 transcription, creating a positive feedback loop in KEAP1 inhibition and NRF2 activation.^51^, ^52^ Consequently, the differential regulation of p62 may be influenced by NRF2 modulation (see Figure 2C,D).

Li et al.^33^ report that CUR inhibits GPX4 and causes ferroptosis by suppressing the PI3K/AKT signaling pathway. Miyazaki et al.^53^ demonstrated that CUR elevates cellular ROS levels, enhancing the pro‐ferroptosis effect by inhibiting both GPX4 and FSP1 levels. Further, our results indicate that CUR contributes to inducing lipid peroxidation and downregulating xCT levels induced by MET in both cell lines treated with COMB (Figure 5), suggesting the promotion of ferroptosis (Figure 5). However, in CT26 cells, GPX4 upregulation after MET and COMB appears to be xCT‐independent, requiring further investigation. As previously discussed, cellular stress leads to NRF2 nuclear translocation and enhances the transcription of antioxidant genes, including GPX4.^54^ Therefore, the observed increase in GPX4 levels may result from NRF2‐mediated antioxidant response activation, suggesting a potential link between NRF2 activity and elevated GPX4 expression in response to combined treatment with MET and CUR.

NF‐κB, a master gene regulator, governs the expressions of genes related to proliferation, cell death, redox reactions, and invasiveness. Figure 6 demonstrates that both CUR and COMBs effectively reduced NF‐κB expression in both cell lines, whereas the MET group did not exhibit this effect. These findings align with previous research showing CUR‐mediated NF‐κB downregulation in CT26 cells.^55^ Metastasis is a major contributor to CRC‐related mortality.^56^ Our study observed that CUR had differential effects on migratory inhibition in CT26 and HCT116 cells, as depicted in Figure 7. In CT26 cells, CUR inhibited migration only within the first 12 h, with wound closure comparable to the CTRL group by 24 h. Conversely, CUR resulted in a sustained migratory inhibition for at least 24 h in HCT116 cells. Notably, the COMB caused substantial migratory inhibition in both cell lines, particularly pronounced in HCT116 cells due to the significant cell death. Other studies have also reported that combining CUR and MET represses cell migration in gastric cancer and hepatocellular carcinoma.^57^, ^58^ Hermeking et al.^16^ demonstrated that CUR suppresses metastasis by activating the ROS/KEAP1/NRF2 pathway, which aligns with our results in the COMB group (Figure 7).

According to the anti‐tumor efficacy results obtained from CT26 and HCT116 tumor‐bearing mice (Figure 8), the immune response seems to play a lesser role in the current combination strategy. This is evident in the CT26 tumor‐bearing mouse model, where the immune system remains intact, unlike the HCT116 tumor‐bearing mouse model established using BALB/c Nude mice, which lacks a functional immune system. Despite this difference, both models exhibit effective anti‐tumor responses. However, several limitations warrant consideration. First, the intricate interplay between apoptosis and autophagy requires further exploration for a comprehensive understanding of their roles in cancer development. Second, the current study employed intraperitoneal injection; future investigations should assess the efficacy of oral administration to bridge preclinical and clinical contexts. Third, while cell migration was assessed in cell culture, exploring tumor metastasis using an in vivo tumor‐bearing model would provide valuable insights. Lastly, investigating the impact of the COMB on anti‐cancer immunity is crucial, given emerging evidence suggesting ferroptosis may modulate therapeutic outcomes through immune regulation.

## 5 CONCLUSIONS

This study provides evidence that the anti‐cancer potential of MET in CRC could be significantly augmented by combining it with CUR. The combined treatment downregulated DMT1 expression, possibly contributing to the anti‐proliferative effects observed in CT26 and HCT116 cells. Furthermore, this study demonstrates that combining CUR with MET leads to ROS accumulation and oxidative stress, ultimately triggering autophagy and ferroptosis that lead to cell death. Additionally, the COMB also results in a substantial inhibition of cell migration. Notably, our in vivo studies revealed that the COMB demonstrates superior tumor suppression effects compared to individual therapies. Nevertheless, considering the heterogeneity of the two CRC cells on combined treatment‐regulated expression of pAMPK, p62 and GPX4, a further investigation should be performed to confirm these associations.

## AUTHOR CONTRIBUTIONS

Conceptualization, H.‐Y.C. and K.‐C.S.; methodology, H.‐W.C.; validation, H.‐Y.C., H.‐W.C. and K.‐C.S.; formal analysis, H.‐W.C.; investigation, H.‐W.C.; resources, H.‐Y.C.; data curation, K.‐C.S.; writing—original draft preparation, H.‐W.C.; writing—review and editing, H.‐Y.C. and K.‐C.S.; visualization, H.‐Y.C. and H.‐W.C.; supervision, H.‐Y.C. and K.‐C.S.; funding acquisition, H.‐Y.C. and K.‐C.S. All authors have read and agreed to the published version of the manuscript.

## FUNDING INFORMATION

This research was supported by a grant from the National Science and Technology Council, Grant No.: MOST 109‐2314‐B‐010‐006‐MY2 (H.‐Y.C.).

## CONFLICT OF INTEREST STATEMENT

The authors declare no conflict of interest.

## ANIMAL STUDY APPROVAL

The animal study protocol was approved by the Institutional Animal Care and Use Committee of National Yang Ming Chiao Tung University (No. 1101012, 1111010).

## Supporting information


Figure S1
Figure S2
Table S1

## Data Availability

The data generated and/or analyzed during the current study are available from the corresponding author upon reasonable request.
