# Supplementary material for: Suppression of colorectal cancer growth: Interplay between curcumin and metformin through DMT1 downregulation and ROS‐mediated pathways
Source: Biofactors. 2024 Nov 28;51(1):e2137. doi: 10.1002/biof.2137 (PMC11681316; doi:10.1002/biof.2137)
Supplement: Supplementary file 1 — Figure S1 Figure S2 Table S1 [file BIOF-51-0-s001.docx]

**Supplementary Materials:**


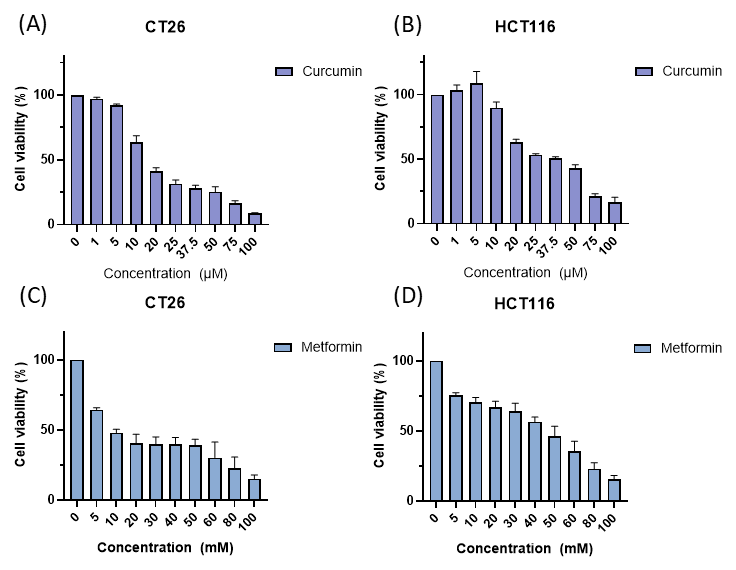


**Figure S1. Cytotoxicity of curcumin and metformin in CT26 and HCT116.** (A-B) Cell viability results of curcumin were determined by MTT assay in CT26 and HCT116, respectively. (C-D) Cell viability results of metformin were determined by MTT assay in CT26 and HCT116, respectively. Error bars indicate SEM and the range of three biological replicates.


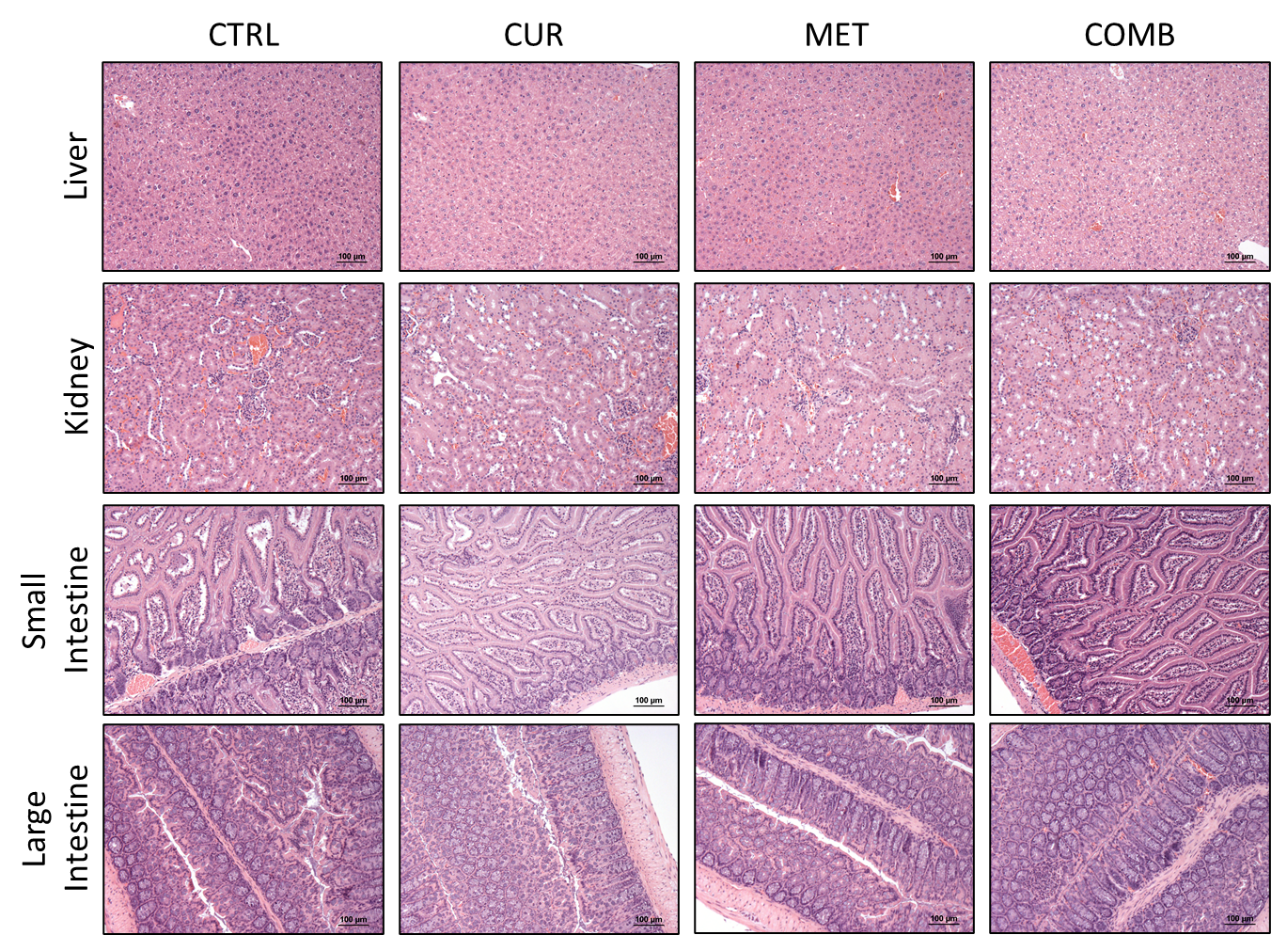


**Figure S2. In vivo toxicity and safety evaluation of combining curcumin with metformin.** H&E staining of liver, kidney, small intestine, and large intestine tissues. Scale bar = 100 μm. All samples were obtained from Balb/c mice.

**Table S1. Information about antibodies**.

| Antibody | Company | Cat. no | Dilution |
| --- | --- | --- | --- |
| β-actin | Genetex | GTX109639 | 1:10000 |
| DMT1 | Cell Signaling | 15083T | 1:1000 |
| phospho-AMPKα (AMPKα) | Genetex | GTX52341 | 1:1000 |
| phospho-AKT (pAKT) | Abclonal | A11016 | 1:1000 |
| mTOR | Genetex | GTX101557 | 1:1000 |
| Cyclin D1 | Cell Signaling | 2978S | 1:1000 |
| NRF2 | Cell Signaling | 12721T | 1:1000 |
| KEAP1 | Cell Signaling | 8047T | 1:1000 |
| Bax | Genetex | GTX109683 | 1:2000 |
| Bcl-2 | Arigo | ARG55188 | 1:1000 |
| Cleaved caspase-3 | Cell Signaling | 9662S | 1:1000 |
| p62 | Genetex | GTX100685 | 1:1000 |
| LC3B | Genetex | GTX127375 | 1:1000 |
| xCT | Cell Signaling | 12691T | 1:1000 |
| GPX4 | Cell Signaling | 52455T | 1:1000 |
| NF-κB | Cell Signaling | 8242S | 1:1000 |
| MMP9 | invitrogen | MA5-13199 | 1:1000 |
